# Expression of the Microtubule-Associated Protein MAP9/ASAP and Its Partners AURKA and PLK1 in Colorectal and Breast Cancers

**DOI:** 10.1155/2014/798170

**Published:** 2014-04-30

**Authors:** Sylvie Rouquier, Marie-Jeanne Pillaire, Christophe Cazaux, Dominique Giorgi

**Affiliations:** ^1^Institute of Human Genetics, UPR 1142, CNRS, 141 rue de la Cardonille, 34396 Montpellier, France; ^2^Cancer Research Center of Toulouse, U1037, ERL5294, INSERM, CNRS and University Paul Sabatier, University of Toulouse, 205, route de Narbonne, 31077 Toulouse Cedex, France

## Abstract

*Background.* Colorectal and breast cancers are among the most common cancers worldwide. They result from a conjugated deficiency of gene maintenance and cell cycle control. *Objective*. We investigate the expression of the microtubule-associated protein MAP9/ASAP and its two partners AURKA and PLK1 in colorectal tumors as well as in ductal breast cancers. *Materials and Methods*. 26 colorectal cancer samples and adjacent normal tissues and 77 ductal breast cancer samples from grade I to grade III were collected. Real-time quantitative PCR was used to analyse the expression of MAP9, AURKA, and PLK1. *Results*. Expression of MAP9 is downregulated in colorectal cancer compared to normal tissues (*P* > 10^−3^), whereas those of AURKA and PLK1 are upregulated (*P* > 10^−4^). In ductal breast cancer, we found a grade-dependent increase of AURKA expression (*P* > 10^−3^), while the variations of expression of MAP9 and PLK1 are not significant (*P* > 0.2). *Conclusions*. MAP9 downregulation is associated with colorectal malignancy and could be used as a disease marker and a new drug target, while AURKA and PLK1 are upregulated. In ductal breast cancer, AURKA overexpression is strongly associated with the tumor grade and is therefore of prognostic value for the progression of the disease.

## 1. Background

Colorectal cancer (CRC) is one of the most frequent cancers worldwide with a rate of mortality close to 33% [1–4]. CRC includes various subtypes whose classification is based on anatomopathological characterization and/or gene profiling [5, 6]. Although mutations leading to hereditary/familial forms of CRC are well documented [7, 8], about 75% of CRC are sporadic [9]. The different steps leading to carcinogenesis by accumulation of a number of genetic alterations have been described [10], including mutations and polymorphisms discussed by Sameer [9]. Numerous studies have shown that genetic instability, mutation, or misexpression of genes involved in genome cell cycle supervision (DNA replication, DNA damage response, mitosis, and checkpoints) is involved from the earlier steps in the process of cell division and proliferation. A number of these genetic defects are associated with CRC [5, 11] and can be efficiently used as biomarkers for prognosis [12]. Using a CGH approach, Orsetti and colleagues [13] characterized genomic instability in colorectal tumors very recently.

Similarly, breast cancer, the most common cancer in women, is associated with numerous mutations and susceptibility loci as described in [14, 15], a number of which such as BRCA1 and BRCA2 [16, 17] are tumor-suppressor genes and/or involved in the DNA damage response and control of cell cycle [18].

We have recently characterized a novel human microtubule-associated protein (MAP) named ASAP or MAP9 [19]. MAP9 localizes at the mitotic spindle and its misexpression results in severe mitotic defects that lead to aneuploidy and cell death. MAP9 is phosphorylated by the mitotic kinases Aurora A (AURKA) [20] and Polo-like kinase 1 (PLK1) [21] to ensure bipolar spindle assembly and centrosome integrity. We have also shown that, in response to DNA damage, MAP9 interacts with and stabilizes the tumor-suppressor TP53 [22]. Furthermore, we recently demonstrated that a normal Map9 function is required for the MT network to allow the first steps of development to proceed [23].

In the present study, we analyzed 26 colorectal tumors versus adjacent coupled normal tissues from the same patients as well as 77 ductal breast tumors to determine whether the deregulation of MAP9 expression could be correlated with malignancy and therefore could be of prognostic value. The two MAP9 regulation partners AURKA and PLK1 were also analyzed.

## 2. Materials and Methods

Single-stranded cDNA samples issued from RT-PCR reactions of 26 pairs of tumoral colorectal tissues and adjacent normal tissues, each derived from the same patient, were part of the collection described in [5, 13] and were used for quantifying AURKA, PLK1, and MAP9 transcripts by real-time PCR. Relative expression levels of each target gene were normalized using the QBase software [24], to four housekeeping control genes (18S, GAPDH, HPRT, and YWHAZ) whose expression levels were previously described as stable [5]. After normalization, results were expressed for each patient as means ± SD and as a ratio tumoral/normal. Briefly, PCR were done in triplicate in 96-well plates in a final volume of 10 *μ*L, using the SYBR Green I Master reaction mix (Roche) on a 480-Light Cycler instrument (Roche). PCR conditions were performed with an initial denaturation of 5 min at 95°C followed by 42 cycles (95°C 20 sec; 56°C 15 sec; 72°C 15 sec) using 1 ng of cDNA template per reaction. Primers were as follows: MAP9 (or ASAP, microtubule-associated protein 9, AY690636), fwd 5′-GCCCTCCAAGCAGAACTGTG-3′, rev 5′-TCAGCAGGAGTGTCTGGCATT-3′; AURKA (NM198433, Aurora kinase A), fwd 5′-TTGGGTGGTCAGTACATGCTC-3′, rev 5′-GTGAATTCAACCCGTGAT-3′; PLK1 (NM005030, Polo like kinase 1), fwd 5′-ACATACCGCCTGAGTCTCCTG-3′, rev 5′-CGCGGGAGCCAACCAGT-3′; HPRT (NM000194, hypoxanthine phosphoribosyltransferase 1), fwd 5′-GGACAGGACTGAACGTCTTGCT-3′; rev 5′-AAAGAATTTATAGCCCCCCTTGA-3′; YWHAZ (NM003406, tyrosine 3-monooxygenase/tryptophan 5-monooxygenase activation protein, zeta polypeptide): fwd 5′-ACTTTTGGTACATTGTGGCTTCAA-3′, rev-5′CCGCCAGGACAAACCAGTAT-3′; GAPDH (NM002046, glyceraldehyde-3-phosphate dehydrogenase), fwd 5′-GAGTCAACGGATTTGGTCGT-3′, rev 5′-GACAAGCTTCCCGTTCTCAG-3′; 18S (ribosomal protein S18), fwd 5′-TTCGGAACTGAGGCCATGAT-3′, rev 5′-TTTCGCTCTGGTCCGTCTTG-3′.

For breast tumors, RT-PCR from 77 ductal tumors were obtained from the Fédération Nationale des Centres de Lutte Contre le Cancer (FNCLCC/Unicancer, cohort PACS01 as already described [18]) and were analyzed using the same protocols except that relative expression of each target gene was normalized to control genes HMBS and IPO8 as previously described [18]: HMBS (NM000190.3, hydroxymethylbilane synthase, fwd 5′-CGCATCTGGAGTTCAGGAGTA-3′, rev 5′-CCAGGATGATGGCACTGA-3′), IPO8 (NM006390.2, importine 8, fwd 5′-GTGTACACACTGGCAGAGCAC-3′, rev 5′-GCCTCCCTGTTGTTCAATCT-3′).

Statistical analyses were performed using the paired *t*-test from GraphPad Prism 6 (http://www.graphpad.com/) for the colon data and the Student's *t*-test for the breast data. Differences were considered significant when *P* < 0.05.

## 3. Results

### 3.1. Mitosis Genes MAP9, AURKA, and PLK1 Are Deregulated in Colorectal Cancers

AURKA and PLK1 are upregulated in a number of cancers such as breast, oesophageal, and colorectal cancers (for review see [25, 26] and references therein). We have analyzed the expression of the 3 mitosis genes AURKA, PLK1, and MAP9, by real-time PCR of 26 coupled primary colorectal carcinomas at different tumoral stages (Table 1 and additional Supplementary Table 1 in Supplementary Material available online at http://dx.doi.org/10.1155/2014/798170). This cohort comprises 26 patients with microsatellite-negative tumors as previously described [5, 13].

**Figure 1:**
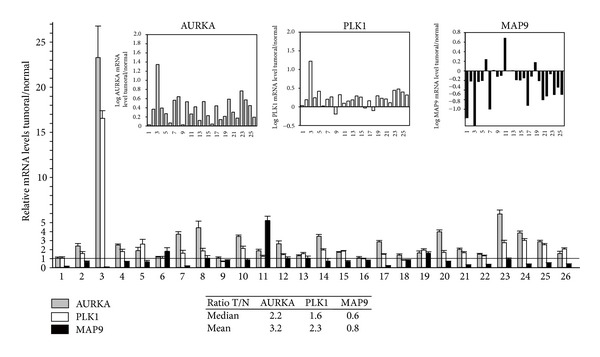
MAP9, AURKA, and PLK1 mRNA levels in colorectal cancer. The mRNA levels were measured by real-time PCR from RT-PCR reactions of 26 colorectal tumors and adjacent normal tissue (numbered 1 to 26 on the *x*-axis). Individual values were normalized to four control genes. Results are expressed as relative mRNA levels (ratio tumor/normal tissue, T/N). The horizontal line for a ratio = 1 indicates the limit for under- or overexpression of the three genes in the 26 tumor samples. In the insets, a logarithmic representation of the mRNA levels of the 26 samples illustrates MAP9 underexpression and AURKA and PLK1 overexpression. A table (inset) recapitulates the arithmetic means and medians of the T/N ratios for the three genes. Differences of gene expression between tumors and normal tissues are statistically significant with *P* < 0.001 for the 3 genes.

In this cohort (Figure 1), AURKA is overexpressed in all the tumors by 2- to 3-fold on average (*P* < 0.0001) with some T/N values as high as 5 and one >20. PLK1 is also upregulated with an average T/N value of ~2 (*P* < 0.0001). Conversely, MAP9 expression is the inverse of the expression of its 2 partners, so that it is downregulated (average T/N ratio ~0.6–0.8) with some T/N ratio as low as 0.04 to 0.1. For example, tumor 3 which strongly overexpresses AURKA and PLK1 (20- and 17-fold, resp.) shows the lowest MAP9 underexpression (~1/25 to that of the normal tissue). A logarithmic representation (insets in Figure 1) illustrates up-/downregulation of the 3 genes in each of the 26 tumors and highlights the strong relative decrease of MAP9 expression. This drop is highly significant (*P* < 0.001) and confirms our previous data showing that ASAP/MAP9 protein expression is highly decreased in the colon cell lines tested [27]. Nevertheless, despite the fact that there is some heterogeneity in the level of expression of these 3 genes in tumors, we can draw a general scheme in which MAP9 is underexpressed and AURKA and PLK1 are overexpressed, even if a few tumors do not strictly display these features. However we did not find (not shown) any correlation between the level of MAP9 expression and the tumor stages (pT1 to pT4 as described in Table 1, *P* > 0.3). Therefore, MAP9/AURKA or MAP9/PLK1 ratios might be valuable hallmarks of CRC.

### 3.2. MAP9, AURKA, and PLK1 Expression in Breast Cancer

We used a subset of primary tumors from French primo-diagnosed patients not treated by neoadjuvant therapy, who represented a subset of women enrolled in an adjuvant multicentric phase III clinical trial (PACS01 trial) [18, 28]. The characteristics of the patients and the results of this clinical trial have been published [28]. Among the 102 patients suffering from breast carcinoma, we chose to focus on the 77 patients (Table 2) who displayed a ductal carcinoma, the other 25 cases being dispersed in lobular and other carcinoma types. Ductal carcinoma comprises ductal carcinoma* in situ* (DCIS) and invasive ductal carcinoma (IDC), the latter representing ~80% of breast cancers. As shown in Table 2, all the 77 patients have positive axillary lymph nodes (1–18) and therefore are IDC patients.

**Figure 2:**
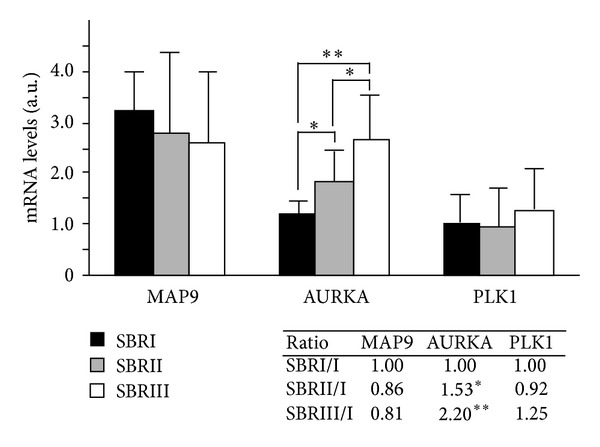
MAP9, AURKA, and PLK1 mRNA levels in ductal breast cancer. The mRNA levels were measured by real-time PCR from RT-PCR reactions of 77 ductal breast tumors. Individual values were normalized to two control genes. Gene expression of MAP9, AURKA, and PLK1 was evaluated by comparing the arithmetic means of the samples belonging to each of the 3 tumor grades SBRI (*n* = 5), SBRII (*n* = 28), and SBRIII (*n* = 44). For MAP9 and PLK1, the differences between the 3 tumor stages were not statistically significant (n.s.), whereas for AURKA there is an increase of gene expression from stage I to III (*P < 0.005, **P < 0.0001). The ratio of gene expression between the 3 tumors stages is indicated in the inset.

Tumor samples were histologically graded SBRI to III as reflecting the severity of the disease (Scarff Bloom Richardson (SBR) grade) [15, 29, 30]. Since coupled normal biopsies are not available in breast cancers, we compared here the 3 tumor stages to each other to investigate whether gene expression could be correlated with the severity of the disease, rather than to compare gene expression in tumors with that of unrelated normal breast samples. As shown in Figure 2, expression of MAP9 and PLK1 remains stable whatever the stage is, whereas the expression of AURKA clearly increased from grade I to III (from 1 to 2.2, inset Figure 2, *P* < 0.001). We then confirmed that AURKA expression is enhanced in ductal breast tumors and is a valuable marker of the evolution of the disease.

## 4. Discussion

In this study we have analyzed the expression of MAP9/ASAP and its two partners AURKA and PLK1 in colorectal and breast tumors. MAP9 is a microtubule-associated protein whose function is crucial for mitosis. AURKA or PLK1 overexpression is associated with spindle defects and aneuploidy, hallmarks of malignant transformation that have been also observed when MAP9 is misexpressed [19]. We show here that, in colorectal tumors, MAP9 is strongly underexpressed whereas AURKA and PLK1 are overexpressed. It was known that the two kinases AURKA and PLK1 were upregulated in a number of tumors including colorectal and breast cancer, as a result of perturbations in centrosome function and spindle assembly that could promote tumorigenesis by enhancing genome instability [25, 26, 31, 32]. Indeed, it has also been observed that overexpression of AURKA in breast, colorectal, and other cancers is associated with the amplification of the corresponding chromosomal region 20q13.2 [33–35]. Similarly, overexpression of PLK1 in tumors is associated with the amplification of the chromosomal region 16p12.2, as revealed by comparative genomic hybridization (CGH) analysis [15]. In this CGH analysis the authors show that, indeed, in breast cancer, there is amplification of the 20q and 16p regions (AURKA and PLK1) whereas the 4q region is often deleted, indicating that downregulation of MAP9 (4q32.1) could be the result, at least in part, of a gene loss. During the submission of this paper, Orsetti and colleagues [13] published a work about CGH analysis of colorectal tumors. This study includes the cohort that we studied in the present work. They show that colorectal tumors accumulate genomic instability (fraction of the genome involved in copy number alterations and number of breakpoints) although this accumulation permits us only to differentiate tumoral and normal tissues and does not correlate with the tumor stages. In parallel to our observations about gene expression, they also show a heterogeneity of genomic alterations between tumors. However, they underline that genomic instability signs colon tumorigenesis with typical patterns of chromosome gains or losses. For example high level gain was observed at 20q where AURKA is located, frequent gain at 16p (PLK1), and loss at 4q (MAP9) in a number of colorectal tumors. In summary, genomic instability, that is, gene gains and losses, correlates with up- and downregulation of MAP9 and its two partners and could be a characteristic of colorectal and breast tumors. However, in CRC we compared expression in tumors with adjacent normal tissues, data then being free from interindividual variability, whereas in ductal breast cancer, adjacent normal tissues were not available. Our study revealed that AURKA is a* bona fide* marker of the severity of the disease whereas PLK1 and MAP9 expression does not correlate with the tumor grade. Nevertheless, NCBI GEO data sets (http://www.ncbi.nlm.nih.gov/geoprofiles/) reveal that MAP9 and PLK1 expression is enhanced ~2 times (GDS 3853) in breast tumors with respect to normal tissue, and AURKA is upregulated (~×20–30). While this observation is in accordance with 16p and 20q amplification, it does not fit with deletion of the MAP9 4q region. In parallel, GEO data set analyses (GDS 2947) of colorectal tumors and adjacent normal tissue show downregulation of MAP9 and upregulation of AURKA and PLK1, with values that are similar to what we observed in this study.

We show here that, in contrast to the two kinases, MAP9 is downregulated in colorectal tumors. This underexpression could be in part the result of chromosome 4q deletion but also the consequence of multiple regulation loops that are deregulated in cancer cells. It is also possible that the overexpression of AURKA and PLK1, two kinases that phosphorylate MAP9 [20, 21], may participate in a feedback regulation of MAP9 expression. Therefore, although MAP9 function is essential for microtubules (MT) in normal cells and is involved in a number of MT-based functions (cytoskeleton, mitosis, and development) it is difficult to decipher whether its underexpression is part of the cause or the consequence of tumorigenesis. It is possible that the perturbation of MAP9 homeostasy may participate in the phenotype of cancer cells and that its expression in these cells is still sufficient to allow mitosis to proceed, or that other pathways may overcome MAP9 deficiency. Nevertheless, this suggests that underexpression of MAP9 might be of pathogenic and prognostic importance, so that this protein might have potential as a new tumor marker for colorectal cancers even though its expression does not correlate with tumor progression and as a drug target for development of new therapies. Indeed, large numbers of inhibitors for polo-like kinases (PLK) and aurora kinases have been developed and are used as anticancer drugs [25]. As cancers are associated with cell proliferation, therapies that focus on the process of cell division have been designed with success [25, 36], and some of these drugs target the microtubule network of the mitotic spindle. Despite the fact that the use of microtubule disruptors might overcome secondary effects on normal cells, MAP9 might be considered as a potential new target for anticancer therapies and a marker of colorectal malignancy.

## Supplementary Material

Supplementary Table 1: Clinical and histological characteristics of CRC patients. Sample numbers (see Figure 1) and corresponding anonymous names in the PACS01 cohort are indicated. For each patient, gender, age, tumor localisation, tumor stage and nodal status (positive or negative) are precised.

## Figures and Tables

**Table 1:** Clinical/histological characteristics of the patients presenting with a colorectal tumor.

Characteristics		No. of patients	%
Sex			
Male		17	65
Female		9	35
Age (median)	73.3		
Interquartile range	12.20		
Range	60.1–89.3		
Tumor (T) stage			
pT1		1	4
pT2		6	23
pT3		12	46
pT4		7	27
Nodal (N) status			
Negative		12	46
Positive		14	54
Distant metastasis (M)			
None detected		16	62
Present		10	38
Overall survival after 3 years		17	65

**Table 2:** Clinical/histological characteristics of the patients presenting with a ductal breast carcinoma.

Characteristics		No. of patients	%
		77	
Age (median)	52		
Interquartile range	11		
Range	34–64		
Tumor (T) stage			
SBRI		5	6.5
SBRII		28	36.4
SBRIII		44	57.1
Positive nodes			
1–3	48		
>3	29		
Overall survival after 3 years		70	91
