# Supplementary material for: Expression of the Microtubule-Associated Protein MAP9/ASAP and Its Partners AURKA and PLK1 in Colorectal and Breast Cancers
Source: Dis Markers. 2014 Apr 30;2014:798170. doi: 10.1155/2014/798170 (PMC4022107; doi:10.1155/2014/798170)
Supplement: Supplementary file 1 — Supplementary Table 1: Clinical and histological characteristics of CRC patients. Sample numbers (see Figure 1) and corresponding anonymous names in the PACS01 cohort are indicated. For each patient, gender, age, tumor localisation, tumor stage and nodal status (positive or negative) are precised. [file 798170.f1.pdf]

Sample nb	Sample Name	Gender	Age	Tumor Localisation	Tumor stage	Nodal status
1	P02.7757	M	62	RightColon	3	-
2	P02.15882	M	89	RightColon	3	-
3	P99.10207	F	76	Rectum	2	+
4	P99.13058	M	67	Rectum	3	+
5	P99.4796	F	60	LeftColon	3	+
6	P99.15422	M	75	RightColon	3	+
7	P99.6928	F	66	Rectum	2	-
8	P99.15220	M	81	LeftColon	1	-
9	P99.7772	F	78	LeftColon	3	-
10	P99.11260	M	82	LeftColon	2	-
11	P99.11612	M	76	Rectum	4	-
12	P99.15390	M	80	Rectum	4	+
13	P99.15773	M	71	LeftColon	2	+
14	P99.8484	M	77	RightColon	3	+
15	P00.788	M	70	RightColon	4	+
16	P00.884	M	67	RightColon	4	+
17	P00.976	F	61	Rectum	3	+
18	P00.2326	F	64	Rectum	3	+
19	P00.5564	M	64	Rectum	4	-
20	P00.2404	M	63	LeftColon	4	-
21	P00.6457	F	75	Rectum	3	+
22	P00.347	F	75	LeftColon	4	+
23	P00.2646	M	60	RightColon	2	-
24	P00.4234	F	77	Rectum	2	-
25	P00.4985	M	72	LeftColon	3	-

26	P00.6365	M	76	Rectum	3	+

Additional Table 1

CRC patients and sample characteristics
